# IPSO-LSTM hybrid model for predicting online public opinion trends in emergencies

**DOI:** 10.1371/journal.pone.0292677

**Published:** 2023-10-10

**Authors:** Guangyu Mu, Zehan Liao, Jiaxue Li, Nini Qin, Ziye Yang

**Affiliations:** 1 School of Management Science and Information Engineering, Jilin University of Finance and Economics, Changchun, China; 2 Key Laboratory of Financial Technology of Jilin Province, Changchun, China; Univerzitet Singidunum, SERBIA

## Abstract

When emergencies are widely discussed and shared, it may lead to conflicting opinions and negative emotions among internet users. Accurately predicting sudden network public opinion events is of great importance. Therefore, this paper constructs a hybrid forecasting model to solve this problem. First, this model introduces an improved inertia weight and an adaptive variation operation to enhance the Particle Swarm Optimization (PSO) algorithm. Then, the improved PSO (IPSO) algorithm optimizes the parameters of the Long Short-Term Memory (LSTM) neural network. Finally, the IPSO-LSTM hybrid prediction model is constructed to forecast and analyze emergency public opinion dissemination trends. The experimental outcomes indicate that the IPSO-LSTM model surpasses others and has high prediction accuracy. In the four emergency predictions we select, the MAPE value of IPSO-LSTM is 74.27% better than that of BP, 33.96% better than that of LSTM, and 13.59% better than that of PSO-LSTM on average. This study aims to assist authorities in quickly identifying potential public opinion crises, developing effective strategies, and promoting sustainable and positive growth in the network environment.

## Introduction

Nowadays, more and more people obtain information and express views using search engines, social media, and other network platforms. Due to social media’s vast and swift diffusion, emergency events are often instantly conveyed to every audience. When the public extensively discusses emergencies, the more negative opinions they express, the higher chances of vicious development of public opinion. Different types of public opinion crises can arise. Internet rumors may cause people to repost more comments on false information [1]. The spread of some sensitive events can also cause collective panic [2]. Additionally, the rapid spread of negative comment posts may distort the truth, inflame emotions and erupt a dangerous situation [3]. Therefore, forecasting and monitoring the public opinion trend is beneficial for government departments to clarify and positively guide the subsequent events.

To steer events in a positive direction, we must pinpoint crucial points in forming public opinion, understand its trajectory, and predict the evolution of emergencies. The research methods related to prediction contain traditional statistics and machine learning [4]. The statistical forecasting methods include the ARIMA [5], the Differential Equation [6], and the Linear Regression [7] et al. While the ARIMA model is straightforward, it requires stable data to generate accurate predictions. If sudden factors cause remarkable fluctuations, the model’s accuracy may suffer. Then, the Differential Equation needs to rely on certain assumptions to analyze the internal relations of research contents. However, this method has a significant error in making mid to long-term predictions. Finally, the Linear Regression model is challenging to express complex data well.

As technology develops, machine learning methods are beginning to emerge. The representative prediction models include the Support Vector Machine [8], Random Forest [9], Markov Chain [10], and Grey Prediction [11]. The Support Vector Machine can solve complex problems in high dimensions, but it currently does not have a universal solution for nonlinear issues. The Random Forest algorithm is excellent at handling data with many sizes, but more is needed to deal with classification regression problems. Implementing the Markov Chain algorithm is simple, but its drawback is a limited ability to predict long-term outcomes. Moreover, the Grey Prediction model also has deficiencies. For example, it may struggle when working with non-linear data, and it’s generally more effective when using a small number of samples. Recent research has focused on deep learning models, an essential branch of machine learning. In terms of public opinion prediction, some scholars integrate the convolutional neural network [12], RBF neural network [13], BP neural network [14], and other models to netizens’ emotions, forwarding behaviors, and communication trends. In addition, scholars also apply neural network models to the dissemination of public opinion [15], public opinion reversal [16], and rumor detection [17].

The online public opinion events data show high-dimensional and nonlinear characteristics [18]. It is entirely appropriate to use deep learning methods for forecasting emergencies. BP neural network can learn and predict complex problems’ nonlinear relations. Recurrent Neural Networks (RNN) can deal with sequential changes in data. As the training time and number of network layers continue to rise, RNN may have gradient explosion or gradient disappearance. Otherwise, there will be over-fitting problems. Hochreiter and Schmidhube (1997) first presented a Long Short-Term Memory network (LSTM), a temporal RNN type [19]. LSTM can effectively address the issue of artificially prolonging time tasks and gradient disappearance in RNN [20]. In addition, LSTM can automatically learn the critical features in time series and forecast continuous data. Scholars have explored sentiment analysis [21] and text sentiment classification [22] for public opinion events using LSTM. Thus, it is reasonable to use LSTM in public opinion prediction.

Combining deep learning methods with computer algorithms in hybrid models is a common practice to enhance the accuracy of emergency prediction. Metaheuristic optimization algorithms have attracted great interest because they can optimize models’ parameters. The Particle Swarm Optimization (PSO) algorithm is proposed from the study of bird predation behavior [23]. Ceylan (2021) used Particle Swarm Optimization to optimize the grey model’s parameters to enhance their predictions’ accuracy [24]. Huang et al. (2020) adopted a modified PSO algorithm to improve the backpropagation parameters to promote the model’s capability [25]. Compared with the Genetic Algorithms and Ant Colony Optimization, the PSO algorithm has high calculation efficiency and can quickly converge to a better result when solving complicated optimization issues. In addition, the algorithm can utilize both individual and group information for collaborative search. At the same time, the algorithm can enhance its effectiveness by adjusting its inertia weight, learning factor, population, velocity, and displacement, ultimately solving the problems of weak searching ability and local optimization. In summary, PSO can positively impact the optimization of parameters in the prediction model. Therefore, this paper integrates the PSO algorithm to improve LSTM parameter selection and prediction accuracy.

Here, we can get the motivation for this paper:

1. The deep learning method can accurately predict nonlinear data, and the LSTM model is an excellent choice for studying online public opinion in emergencies.
2. The modified PSO algorithm optimizes the LSTM parameters and improves the trend prediction accuracy in emergencies.

This paper uses deep learning and metaheuristic optimization algorithm to set up a public opinion emergency prediction model. The main research contents are as follows:

Firstly, we innovatively improve the metaheuristic optimization algorithm PSO by introducing modified inertia weight and adaptive mutation operation to generate IPSO.

Secondly, we use the IPSO algorithm to enhance the LSTM’s parameters and construct the IPSO-LSTM public opinion prediction model.

Finally, the four empirical studies of online public opinion emergencies confirm the model’s prediction accuracy and superiority.

The paper is divided into five parts: introduction, literature review, methods and research framework, empirical results analysis and discussion, and conclusions and suggestions.

## Literature review

### Online public opinion

Online public opinion refers to the social issues gathered by the public expressing different views on social platforms. Following public opinion events, individuals may develop new thoughts, viewpoints, emotions, and behaviors. The ideas expressed by people online can reflect and amplify societal issues. In recent years, studies on public opinion have primarily concentrated on analyzing [26], spreading [27], warning [28], and supervising it [29]. Public opinion analysis chiefly discusses network events’ evolution trend [30], topic classification, and the hot topic comments’ emotional tendency detection [31–34]. The research content of public opinion propagation typically includes the following aspects: public opinion events’ development cycle [35], evolution, communication, and influence [36, 37]. Public opinion warning research utilizes computer and artificial intelligence technology to create an early warning model [38–40]. Public opinion supervision formulates regulatory strategies and takes measures for corresponding public opinion events [41]. Analyzing online public opinion during emergencies can help us better understand the transmission and development of events and react quickly to any crisis of general view. In essence, studying such events can aid in our comprehension of the situation.

### Public opinion emergency prediction

According to the Emergency Response Law of the People’s Republic of China, emergencies are sudden events that cause or are likely to cause significant social harm and require emergency response measures, including natural disasters, accidents, public health, and social security events. Public opinion emergency prediction means analyzing public opinion development and emergency events’ topic trends and providing targeted suggestions. Related research can be divided into three areas: emotion prediction, hot topic identification, and trend prediction. First of all, emotion prediction is to study Internet users’ emotional changes during events. Some scholars predict public emotion through text sentiment classification [42]. Other scholars create related corpora to track Twitter users’ feelings and emotions toward new directions and conduct decision-making [43, 44]. These studies assist in directing the emotional choices of internet users strategically. Then, multiple sub-topics will be derived with the continuous spread of emergencies. Related research on hot topic identification is to find netizens’ fundamental and repeated topics in time based on the text information characteristics [45] and identify rumors [46]. Finally, trend prediction is to perceive the overall spread trends of emergencies in advance and to find the best guiding point of events. The research includes predicting the propagation trend of popular opinion [47], the change in public opinion trend [48], and the evolution of social viewpoint [49, 50]. Thus, predicting the popularity trend of emergencies can help relevant departments master public opinion’s development. The authorities can correctly guide the negative emotions of netizens, avoid the vicious influence of events, and increase the capacity to cope with public opinion risks.

### Research on LSTM prediction

The LSTM neural network’s gating mechanism solves gradient disappearance and explosion, its memory unit retains information for extended periods, and parallel computation boosts efficiency. The above advantages make LSTM excellent in processing sequence data and long-term dependencies, leading to its widespread use in prediction tasks. Many scholars also employ different combinations of LSTM variants and hyperparameters to achieve better performance [51, 52]. In recent years, scholars have combined various metaheuristic optimization algorithms to optimize LSTM parameters to improve forecasts’ accuracy. Jovanovic et al. used the Salp Swarm Algorithm to determine the satisfactory parameters of the LSTM model, which performs well in crude oil price prediction [53]. Stankovic et al. introduced the Salp Swarm Algorithm to develop an LSTM model for Ethereum price prediction [54]. Mu et al. combined the metaheuristic algorithm and deep learning to build the MS-SSA-LSTM stock price prediction model [55]. Drewil and Al-Bahadili proposed a model based on the Genetic Algorithm (GA) and LSTM for predicting air pollution [56]. Nebojsa et al. employed the Sine Cosine Algorithm (SCA) to tune hyperparameters for LSTM and GRU models, discovering its potential in energy forecasting [57]. Suddle and Bashir operated metaheuristics to optimize LSTM architecture and improve sentiment analysis accuracy [58]. In conclusion, optimizing LSTM parameters with metaheuristic algorithms can effectively enhance the ability of model prediction.

## Methods and research framework

### Particle swarm optimization algorithm

The PSO algorithm takes inspiration from the collective foraging behavior of birds. By sharing information, the birds can locate the best destination to achieve their goal effectively. The PSO algorithm generates n random particles and assigns them a specific position and speed in D-dimensional space. These particles search for an optimal solution and save it as their personal best. Then, the algorithm finds the best global solution by comparing an individual’s extremum in the PSO with the current optimal extremum. Eqs (1) and (2) renew the particle’s velocity and position.

$$v_{id}^{k+1}=wv_{id}^{k}+c_{1}r_{1}\left(pbest_{id}-x_{id}^{k}\right)+c_{2}r_{2}\left(gbest_{d}-x_{id}^{k}\right)$$ (1)

$$x_{id}^{k+1}=x_{id}^{k}+v_{id}^{k}$$ (2)

Where *i* = 1, 2,…, *N* is the serial number of the particle, *d* = 1, 2,…, *D* represents the serial number of particle dimensions, *w* represents inertia weight, and *c*1 and *c*2 are acceleration coefficients of learning behaviour. *r*1 and *r*2 are uniformly random values ranging from 0 to 1 to make the search more random. *k* represents the number of iterations, and *pbes*_*id*_ is the personal best location of particle *i* in the *d* dimension. $x_{id}^{k}$ and $v_{id}^{k}$ represent the position vector and the velocity vector of particle *i* within the *d* dimension during the *k* iteration, and *gbes*_*d*_ is by far the best position in the group.

### Improved particle swarm optimization algorithm

This paper uses the PSO algorithm to optimize LSTM for prediction because of its advantages. Owing to its strong search capabilities, the PSO algorithm is highly effective in finding solutions to complex problems within the search space. Moreover, the algorithm has a relatively fast convergence speed, which makes it suitable for solving nonlinear optimization problems. Finally, the stability of the PSO algorithm is essential in obtaining precise predictions. While the PSO algorithm is valid, it does have limitations. Specifically, it tends to become stuck in extreme local values and challenges to balance its local and global search capabilities. Below is a detailed improvement procedure to enhance the PSO algorithm.

#### Improve inertia weight

Inertia weight is a crucial factor in particle swarm optimization that determines how particles can retain their motion at the last moment. Adjusting the inertia weight can enhance the algorithm’s performance. A more considerable inertia weight is advantageous for global search, whereas a more minor one benefits local search. Thus, a strategy of inertia weight with a nonlinear decrease is utilized to balance local and global search capabilities. In other words, we add a hyperbolic tangent curve to adjust the change of inertia weight, as shown in Eq (3).

$$w=w_{max}-\left(w_{max}-w_{min}\right)+tanh\left(\frac{t}{4max\_iter}\right)$$ (3)

Where *w*_*max*_ and *w*_*min*_ are the upper and lower bounds of inertia weight, respectively, *t* indicates the iterations, and max_*iter* represents the maximum iterations number. This strategy has a slow decline rate of *w* during the initial phase of the search, which gives particles enough time for global search and avoids falling into local optimum. As the search period progresses and the decline rate of *w* decreases, particles gradually strengthen the local search capability and focus on detailed local search to accurately determine the optimal global solution.

#### Add adaptive mutation operation

This paper introduces an adaptive mutation operation that can improve the algorithm’s ability to avoid getting stuck in a local optimum by adjusting the mutation probability adaptively. By increasing the evolutionary algebra, this operation minimizes variation and helps to get optimal parameters for LSTM. Eq (4) is a specific adaptive mutation operation formula.


prob=0.5⋅t/max_iter+0.5 (4)

Adding an adaptive mutation operation allows the PSO algorithm to judge whether to jump out of the current optimal local position and search in a more extensive solution space. The population’s diversity is maintained when expanding the search scope of solution space. Moreover, adding an adaptive mutation operation can prompt the effectiveness of algorithm search and enhance the population’s capacity to search for the optimal global solution.

### Long short-term memory neural network

**Fig 1:**
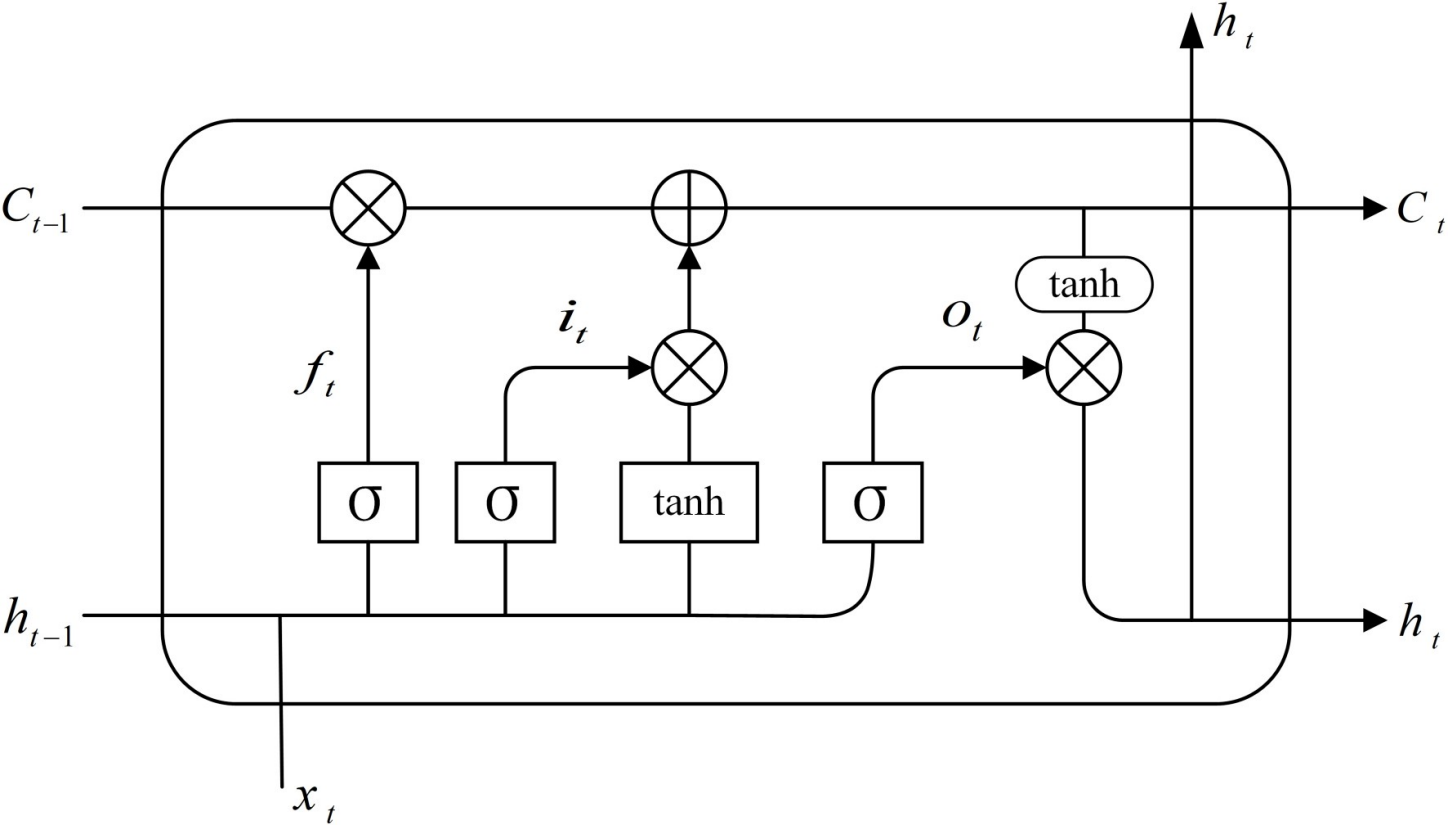
LSTM unit structure.

LSTM neural network can make up for shortcomings of RNN effectively. Compared with RNN, LSTM adds memory units for self-measurement and mainly controls the logical realization by integrating forgetting, input, and output gates. Its unit structure is displayed in Fig 1.

At each point, a corresponding state *C*_*t*_ records the previous information and can ensure the practical preservation of the gradient. The forgetting gate (*f*_*t*_) determines the discarded information in the unit state. *h*_*t*−1_ indicates the last unit output, *x*_*t*_ represents the current unit input, and σ means the sigmoid function.

$$f_{t}=\sigma \left(W_{f}\cdot \left[h_{t-1},x_{t}\right]+b_{f}\right)$$ (5)

Where the sigmoid function is:

$$\sigma \left(c\right)=1/\left(1+e^{-c}\right)$$ (6)

The input gate (*i*_*t*_) decides the amount of new content that shall be included into the unit state. The sigmoid layer identifies the information that needs to be updated, while the tanh layer generates vectors to obtain alternative update information. Then, the unit state is renewed by incorporating the input gate and sigmoid layer.


$$i_{t}=\sigma \left(W_{i}\cdot \left[h_{t-1},x_{t}\right]+b_{i}\right)$$ (7)

$$c_{t}^{\prime }=\text{tanh}\left(W_{c}\cdot \left[h_{t-1},x_{t}\right]+b_{c}\right)$$ (8)

$$c_{t}=f_{t}\cdot c_{t-1}+i_{t}\cdot c_{t}^{\prime }$$ (9)

The hyperbolic tangent function is:

$$\text{tanh}\left(c\right)=\left(e^{c}-e^{-c}\right)/\left(e^{c}+e^{-c}\right)$$ (10)

The output gate (*o*_*t*_) generates the current unit output value and the unit’s condition, and the sigmoid layer runs to confirm the unit state part to be output. Finally, the unit state is handled by the tanh layer to determine the outcome.


$$o_{t}=\sigma \left(W_{o}\cdot \left[h_{t-1},x_{t}\right]+b_{o}\right)$$ (11)

$$h_{t}=o_{t}\cdot \text{tanh}\left(c_{t}\right)$$ (12)

### IPSO-LSTM model construction

**Fig 2:**
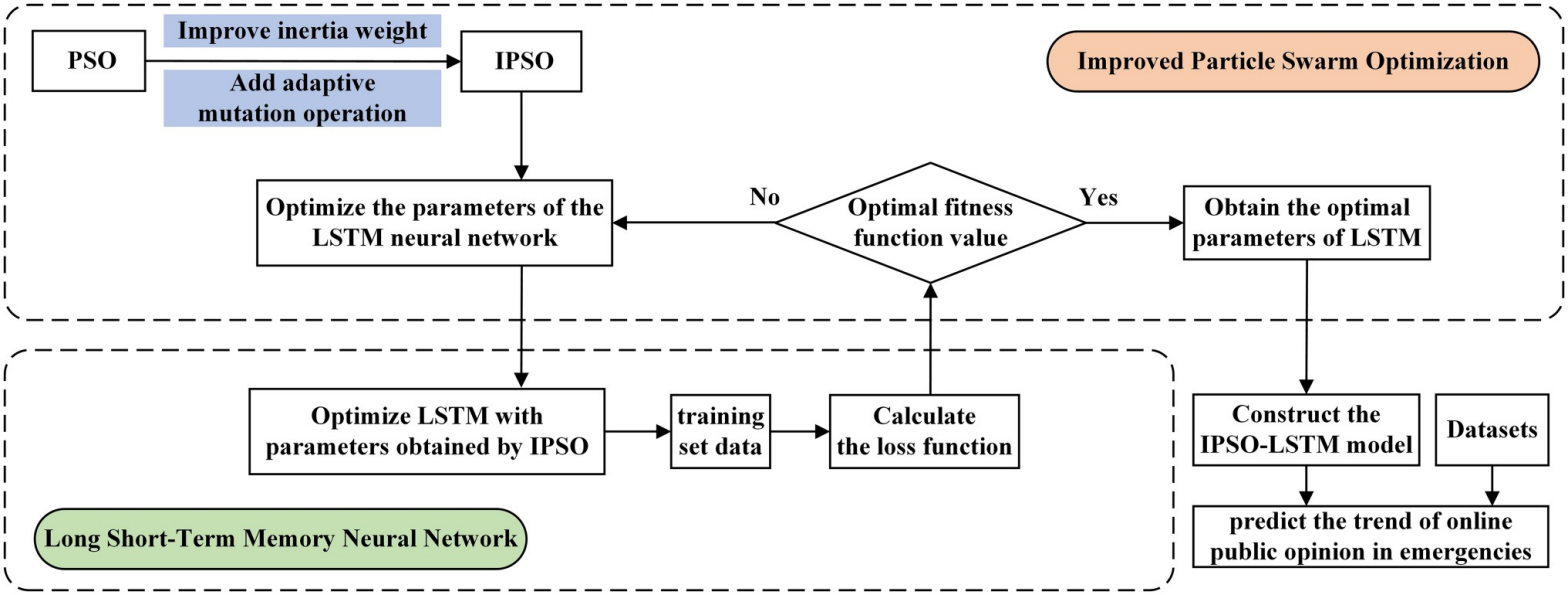
The procedure of the IPSO-LSTM prediction model construction.

The initial value of parameters in LSTM neural networks affects the network’s performance significantly. The LSTM neural network learning is an automatic adjustment procedure. Modifying the thresholds, weights, and initial values will immediately impact convergence, learning time, and local minimum. Therefore, we create the IPSO-LSTM model, which uses the IPSO algorithm to find the optimal initial parameters of LSTM. This method improves the LSTM prediction model’s performance and reduces subjectivity in parameter selection. The parameter settings of the IPSO algorithm are shown in Table 1. The primary construction procedure of the IPSO-LSTM prediction model is shown in Fig 2.

**Table 1:** The parameter settings of the IPSO algorithm.

Parameters	Value
The population number	5
The maximum iteration	10
Dimension	6

The IPSO-LSTM prediction model’s specific steps are as follows:

Step 1: Improve the PSO algorithm. The improved PSO (IPSO) algorithm is formed by introducing an enhanced inertia weight and an adaptable mutation operation.
Step 2: Initialize parameters. Initialize the IPSO and take the LSTM neural network’s parameters to be optimized. Each particle represents a combination of LSTM neural network parameters.
Step 3: Preprocess the data. The dataset is separated into the training and testing sets proportionally.
Step 4: Optimize the fitness function. Take the loss function of LSTM on the training set as the fitness function. According to the minimum loss function principle, initialize each particle search’s best solution and the particle group search’s global best solution.
Step 5: Update particle swarm parameters. Adjust the speed and position parameters for the particle swarm. Moreover, following the fitness function, update the particle swarm’s personal and global best solution.
Step 6: Construct the IPSO-LSTM prediction model. Repeat step 5 until output the best fitness function value, get the optimal LSTM parameter list, and build the prediction model.

### Model evaluation index

The accuracy of prediction is an essential standard to estimate the predictive model’s performance. In this paper, the precision and credibility of the model are evaluated by three indexes, which are Mean Absolute Error (MAE), Root Mean Square Error (RMSE), and Mean Absolute Percentage Error (MAPE). MAE and RMSE represent two indexes for the mean range of prediction error. MAPE is a popular indicator regarded as the percentage of forecast accuracy. The formula for calculating each index is shown from Eqs (13) to (15).

$$MAE=\frac{1}{n}\sum_{i=1}^{n}\left|\left(y_{i}^{\prime }-y_{i}\right)\right|$$ (13)

$$RMSE=\sqrt{\frac{1}{n}\sum_{i=1}^{n}{\left(y_{i}^{\prime }-y_{i}\right)}^{2}}$$ (14)

$$MAPE=\frac{1}{n}\sum_{i=1}^{n}\left|\frac{y_{i}^{\prime }-y_{i}}{y_{i}}\right|$$ (15)

Where n is the sample capacity, and *y*_*i*_ and $y_{i}^{\prime }$ are the actual and predicted values, respectively. Models with lower MAE, RMSE, and MAPE values are generally considered to perform better.

## Empirical results analysis and discussion

### Data acquisition

In this paper, the data on emergencies comes from the platform https://ef.zhiweidata.com/. This platform collects data from China’s well-known social media, likely WeChat, Weibo, Baidu, et al. In addition, the platform aggregates Internet social hot spots and figures out the attention trends of netizens. According to the types of emergencies defined by the Emergency Law of the People’s Republic of China, we select four typical cases for each category and cover different countries. We gain 82361 pieces of information from the "Turkey Earthquake" event between February 6–21, 2023, 25157 pieces of information from the "Changsha building collapse" event in China from April 29, 2022, to May 12, 2022, 88840 pieces of information from the "Sanya epidemic" event in China from August 1, 2022, to September 4, 2022, and 31525 pieces of information from the "South Korea stampede accident" event from October 29, 2022, to November 11, 2022. And through the above four emergencies to verify The IPSO-LSTM model’s ability. The model adopts the rolling prediction method. We take 70% of the data as training and 30% as testing sets.

### IPSO-LSTM prediction model results analysis

This paper uses the IPSO algorithm to find the optimal initial parameters of LSTM. Table 2 shows the specific LSTM parameters obtained from the IPSO algorithm.

**Table 2:** The parameters of LSTM.

Emergencies	LSTM parameters					
	lr	epochs	batch size	hidden1	hidden2	fc
"Turkey earthquake"	0.0065	91	92	10	1	87
"Changsha building collapse"	0.0089	90	91	19	9	55
"Sanya epidemic"	0.0098	24	34	10	10	31
"South Korea stampede accident"	0.0060	94	18	12	6	81

**Note**: lr: learning rate. hidden1: number of nodes in hidden layer 1. hidden2: number of nodes in hidden layer 2. fc: number of nodes in the fully connected layer.

**Fig 3:**
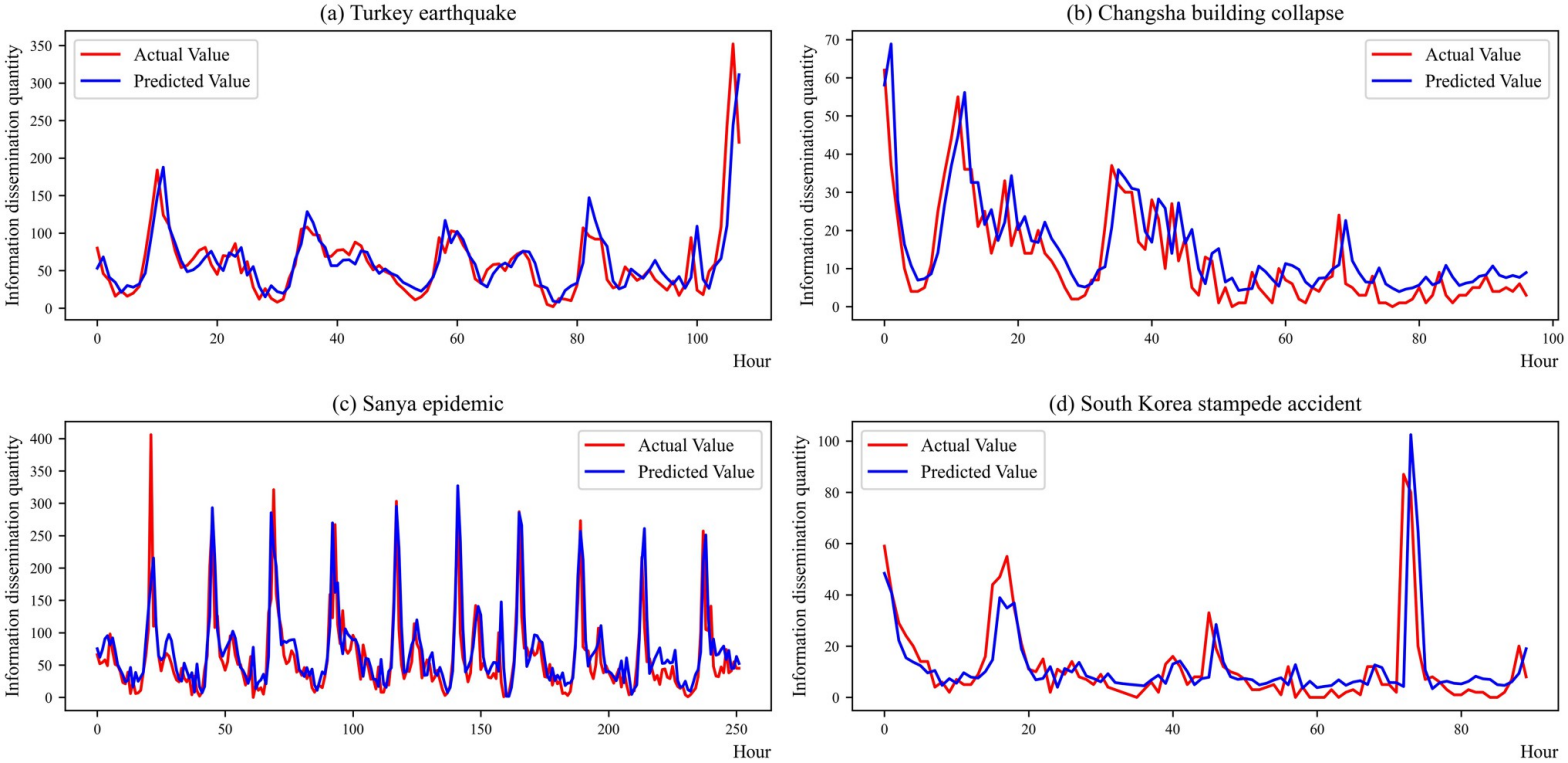
IPSO-LSTM model prediction results. (a) Turkey earthquake; (b) Changsha building collapse; (c) Sanya epidemic; (d) South Korea stampede accident.

The IPSO-LSTM model forecasts the trend based on the four emergencies mentioned. Fig 3 displays the predicted results of the discussion heat trend. The horizontal axis indicates the hour points, and the vertical axis shows the information dissemination quantity of the event. The red line shows the actual values, while the blue line represents the predicted values. Regardless of the type of emergency or the duration of the events, the fitting effect is quite good. Compared with the "Turkey earthquake" and the "Sanya epidemic" events predictions, the other events’ image fitting is relatively poor. One of the main reasons for this is low attention to the incident. Additionally, the duration of heat towards the event is short, and the number of training data available is relatively small.

### Comparative analysis of models

**Fig 4:**
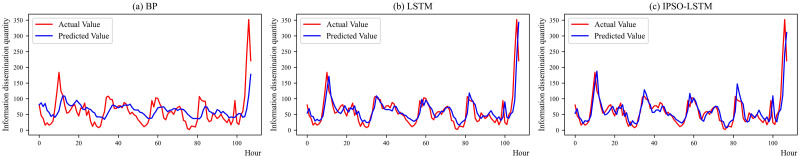
Predictive comparison results of the "Turkey earthquake" event. (a) BP; (b) LSTM; (c) IPSO-LSTM.

**Fig 5:**
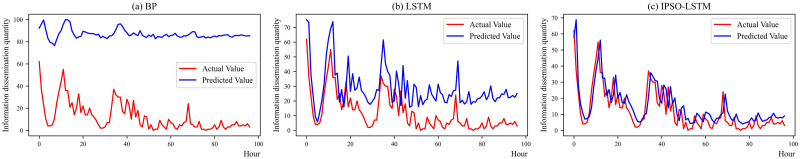
Predictive comparison results of the "Changsha building collapse" event. (a) BP; (b) LSTM; (c) IPSO-LSTM.

**Fig 6:**
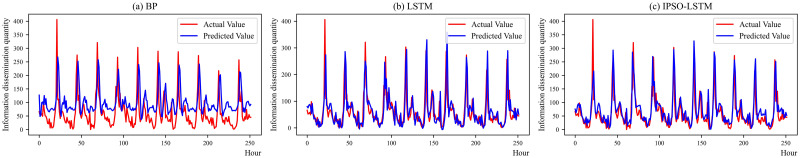
Predictive comparison results of the "Sanya epidemic" event. (a) BP; (b) LSTM; (c) IPSO-LSTM.

**Fig 7:**
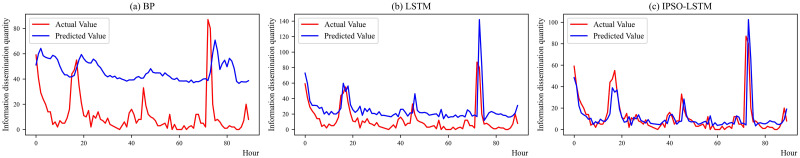
Predictive comparison results of the "South Korea stampede accident" event. (a) BP; (b) LSTM; (c) IPSO-LSTM.

We test the proposed model’s availability using BP and LSTM neural network methods and conduct a contrast experiment with the IPSO-LSTM. Figs 4–7 show the prediction results for each event.

After comparing the prediction models, it is clear that the three neural network models have different forecasting capabilities. The LSTM prediction model is significantly superior to the BP model in fitting precision. Based on the data presented in Figs 5 and 7, the BP fit effect is inferior, and the forecasted situation does not accurately reflect the current state of public opinion. Although there has been a notable enhancement in the accuracy of LSTM fitting, there is still a discrepancy compared to the actual value. In both the "Changsha building collapse" and "South Korea stampede accident" events, the MAPE of the IPSO-LSTM method demonstrates a significant improvement compared to the BP and LSTM methods. Specifically, in the "Changsha building collapse" event, the IPSO-LSTM method outperforms the BP and LSTM methods by 90.11% and 57.71%, respectively. Similarly, in the "South Korea stampede accident" event, the IPSO-LSTM method revealed an improvement of 25.61% and 7.46% compared to the BP and LSTM methods. Unfortunately, online public opinion events are often overlooked in their early stages. Once public opinion reverses or rumors spread, there might need to be more time to implement effective regulatory strategies. The model developed in this paper can be crucial in forecasting the situations mentioned above.

In addition to the above methods, we add the PSO-LSTM model and three machine learning methods, RF, SVR, and XGBoost, to predict and analyze the four events. Table 3 shows the experimental consequences of the different models. The first three of each event in Table 3 belong to machine learning methods, and the last four belong to deep learning ones. The average MAPE values of the machine learning models for the four events are 0.8175, 3.4991, 0.9703, and 1.7912, respectively. Similarly, the average MAPE values of the deep learning prediction models are 0.2940, 2.3797, 0.6737, and 1.0094, respectively. Compared with the average MAPE values of machine learning models, the deep learning models decreased by 64.03%, 31.99%, 30.56%, and 43.64%, respectively. The results show that the deep learning models’ prediction ability surpasses machine learning models. Moreover, in an overall comparison of deep learning models, the MAPE value of IPSO-LSTM is 74.27% better than that of BP, 33.96% better than that of LSTM, and 13.59% better than that of PSO-LSTM on average. Therefore, the proposed IPSO-LSTM model is better than other deep learning models in forecasting public opinion. The MAE and RSME values also have the same trend as MAPE values. The sole distinction lies in the model comparison of the "Changsha building collapse" event, where the MAE value of the IPSO- LSTM model is marginally inferior to that of the PSO-LSTM. However, the effect is minimal, and the RMSE and MAPE values are better. After thorough analysis, we concluded that the IPSO-LSTM model is better and more reliable in forecasting public opinion trends.

**Table 3:** Comparison of prediction models.

Emergencies	Method category	Prediction model	MAE	RMSE	MAPE
"Turkey earthquake"	machine learning	RF	25.2257	34.8145	0.9037
		SVR	21.5524	29.3838	0.6701
		XGBoost	37.3460	51.2644	0.8788
	deep learning	BP	0.1653	0.2451	0.4214
		LSTM	0.1115	0.1730	0.2675
		PSO-LSTM	0.1074	0.1637	0.2534
		IPSO-LSTM	**0.1056**	**0.1604**	**0.2338**
"Changsha building collapse"	machine learning	RF	16.2638	18.3913	4.8947
		SVR	10.1826	11.3569	2.9052
		XGBoost	10.1053	12.2908	2.6973
	deep learning	BP	2.4038	2.4283	6.5351
		LSTM	0.5419	0.5988	1.5286
		PSO-LSTM	**0.1950**	0.2855	0.8086
		IPSO-LSTM	0.2035	**0.2618**	**0.6464**
"Sanya epidemic"	machine learning	RF	30.8463	50.7394	1.0574
		SVR	31.2465	52.8725	0.9118
		XGBoost	31.7900	52.9577	0.9418
	deep learning	BP	0.2675	0.3245	0.8047
		LSTM	0.1519	0.2436	0.7291
		PSO-LSTM	0.1481	0.2165	0.6291
		IPSO-LSTM	**0.1327**	**0.2041**	**0.5317**
"South Korea stampede accident"	machine learning	RF	10.8908	18.2156	2.1984
		SVR	7.9679	14.3025	1.3345
		XGBoost	11.0595	19.7747	1.8407
	deep learning	BP	0.8401	0.8774	1.2012
		LSTM	0.3728	0.4424	0.9656
		PSO-LSTM	0.1521	0.2874	0.9771
		IPSO-LSTM	**0.1481**	**0.2812**	**0.8936**

## Conclusions and suggestions

### Conclusions

This paper combines metaheuristic optimization algorithms with LSTM neural networks and constructs the IPSO-LSTM prediction model to forecast emergency public opinion. We compare the forecast accuracy of deep learning and traditional machine learning models for four types of sudden public opinion events. Furthermore, we also contrast the IPSO-LSTM model with other deep learning models. The experimental results provide solid evidence supporting the superior predictive performance of the IPSO-LSTM model.

The LSTM neural network model can address the issues related to gradient explosion and disappearance. However, it can be challenging to figure out the appropriate parameters when utilizing it for nonlinear and complex data related to online public opinion. The process of selecting parameters for a model depends on the researcher’s experience, which can be arbitrary and impact the accuracy of the model’s predictions. Thus, we choose the metaheuristic optimization algorithms to gain the LSTM model’s parameters. Out of all the optimization algorithms, the PSO algorithm stands out for its low adjustment parameters, high efficiency, robustness, and ease of implementation. In the meantime, we make two improvements to enhance the PSO algorithm and establish the IPSO-LSTM public opinion prediction model. On the one hand, we improve the inertia weight. On the other hand, we add an adaptive mutation operation to overcome the algorithm’s limitations. The improved PSO algorithm can help LSTM automatically search for the best combination of parameters and decrease the subjectivity of manually adjusting parameters. Ultimately, we improve the LSTM model parameters’ global optimization ability, reduce the model’s randomness, and increase its prediction accuracy and stability by enhancing the PSO algorithm.

The IPSO-LSTM prediction model analyzes the public opinion of emergencies in four categories of networks. Based on the results, the model is highly effective and accurate. Specifically, the forecasting precision of events with low initial heat and short duration can also be significantly improved. Through the evaluation results of different events, deep learning models demonstrate excellent prediction performance compared to traditional machine learning models. The IPSO-LSTM prediction model has proven superior in public opinion forecasting based on evaluation indexes such as MAE, RMSE, and MAPE. In addition, the average MAPE value predicted by BP, LSTM, PSO-LSTM, and IPSO-LSTM models for four events is 2.2406, 0.8727, 0.6670, and 0.5763, respectively. The MAPE value of IPSO-LSTM is 74.27% better than that of BP, 33.96% better than LSTM, and 13.59% better than PSO-LSTM. The importance of using the IPSO algorithm in conjunction with the LSTM model is confirmed. In other words, the IPSO-LSTM model can efficiently identify critical nodes of public opinion in emergencies with higher accuracy and speed.

In summary, this paper established that the IPSO-LSTM public opinion prediction model is valid and practical, which can enhance the forecast precision of public opinion trends. Moreover, the model should provide information for the authorities in response to public opinion crises and promote a sustainable and healthy online environment.

### Suggestions

According to the conclusions above, we put forward some suggestions on the network’s public opinion of emergencies.

From the perspective of netizens, the sanity of netizens greatly influences the evolution of emergencies. If internet users refrain from sharing negative emotions, avoid unthinkingly following others’ opinions, and instead maintain a rational and observant approach, they can fundamentally reduce the explosion of online public opinion. On the contrary, if netizens become uncertain about negative and untrue information, they may unintentionally spread misleading news that could worsen the severity of emergencies. Therefore, it is essential to encourage internet users to monitor their language and actions, express themselves thoughtfully, and contribute to managing public opinion crises.

From the perspective of social platforms, each event’s information data and user behavior need to be monitored. A widespread outbreak of online public opinion can result in conflicting views, negative attitudes, rumors, and public opinion crises. Additionally, it can cause social media platforms to become overloaded with information processing. Adequate real-time supervision is essential for the platform to maintain control over events, promptly address potential public opinion crises, and take necessary actions to ensure the network environment continues to thrive. It is also vital to capture the emotional expression of users, which can assist the platform in grasping the ideological dynamics of netizens.

From the perspective of government regulatory authorities, it is crucial to predict the public opinion emergency trend, supervise the fluctuation of events, and swiftly intervene and guide as needed. Once an emergency causes a wide range of negative information dissemination, it may lead to the spread of rumors and fall into a public opinion crisis. If the government fails to respond to the spread of rumors, it may cause online public opinion to get out of control. In China, every city government has set up an online public opinion monitoring office. If the government adopts the IPSO-LSTM model constructed in this paper, authorities can effectively anticipate online public opinion trends and identify potential risks. Accordingly, the government can obtain the actual situation of the development during crises and take appropriate measures to avoid potential hazards such as rumors. In the necessary time, they can directly disclose factual information to prevent Internet users’ excessive speculation. In addition, government regulatory authorities should build up the risk response mechanism, timely update the risk level of events, and measure both current and long-term risks to strengthen the monitoring and response abilities of uncertain public opinion events.

### Future prospects

Although the model has excellent precision in forecasting the network public opinion on emergencies, netizens’ sentiments and comment-forwarding behaviors, such as the number of likes, forwards, and reviews posted on the web, can be included to achieve preferable predictions in future research.

The neural network method has better accuracy in predicting network public opinion in emergencies and can effectively and timely discover potential public opinion crises. We can make further improvements to optimize the monitoring of emergencies continuously. For instance, we should select a novel algorithm to optimize the model’s parameters or an enhanced neural network model for prediction.
